# Editorial

**DOI:** 10.7705/biomedica.7930

**Published:** 2025-03-28

**Authors:** Julián Alfredo Fernández-Niño

**Affiliations:** 1 Subsecretario de Salud Pública, Secretaría Distrital de Salud de Bogotá, Bogotá, D. C.; profesor, Universidad del Norte, Barranquilla jafernandeznino@gmail.com Universidad del Norte Subsecretario de Salud Pública Secretaría Distrital de Salud de Bogotá Universidad del Norte Bogotá, D. C. Barranquilla Colombia jafernandeznino@gmail.com

## Migración y salud en la época de los nacionalismos

El panorama político global actual representa uno de los escenarios más desafiantes para las políticas migratorias observado en décadas recientes ^1^. En varios países de altos ingresos, como Estados Unidos y el Reino Unido, aunque también en países de bajos y medianos ingresos, como Argentina, el discurso antiinmigrante ha resurgido como una estrategia electoral efectiva, replicando narrativas exitosas del pasado. Estas narrativas se fundamentan en ideas recurrentes: los migrantes son considerados una amenaza para la democracia, una carga para los sistemas sociales - especialmente los de salud y asistencia social-, una competencia injusta dentro del mercado laboral, una fuente de enfermedades y, el argumento más reiterado últimamente, una amenaza para la seguridad pública ^2^. Estos discursos, carentes de evidencia, no solo favorecen el estigma y la discriminación contra los migrantes, sino que también dificultan el avance en la garantía de sus derechos.

Detrás de estas posiciones, que hoy recobran tanta fuerza, subyace una lógica nacionalista reemergente que choca frontalmente con los objetivos de cooperación global. Los gobiernos de los países que adoptan estas posturas consideran que su primera -o incluso única- obligación es protegerse a sí mismos, a sus propios ciudadanos y que las obligaciones con sus otros habitantes son cargas indeseables que deben reducirse. Bajo esta perspectiva, se delega la responsabilidad de los derechos de los migrantes a los países de origen, al tiempo que se le atribuye a la migración un impacto negativo sobre la seguridad, la estabilidad económica e incluso la cohesión social de las naciones receptoras ^3^. Estas narrativas permiten -como ocurrió justo antes de la segunda guerra mundial- que se tenga un chivo expiatorio para la crisis de la democracia y los cambios económicos desfavorables en esos países destino.

Esta situación, junto con el continuo debilitamiento de los mecanismos multilaterales internacionales, amenaza seriamente los objetivos de desarrollo humano, que dependen de la cooperación y la inclusión económica, social y cultural de los migrantes. También, es incompatible con una visión de los derechos de los migrantes que transcienda las fronteras ^4^, un esfuerzo anhelado y perseguido por varias generaciones. Las fallas, contradicciones y sombras de la cooperación internacional no deberían llevarnos a la conclusión de que la fragmentación nacionalista es la solución, al menos si queremos asumir, como humanidad, las luchas comunes contra la pobreza, el hambre, la mortalidad materna e infantil y las enfermedades infecciosas. Más ampliamente, el renunciar a considerar la acción global como necesaria para reducir las inequidades en salud -por definición, injustas y evitables- puede llevar no sólo a retroceder décadas en el consenso internacional, sino a que dichas brechas se incrementen, afectando a las personas más pobres y, dentro de ellas, a las mujeres, niños, niñas y adolescentes.

Las políticas de migración y salud están profundamente interconectadas con las políticas migratorias en general. Por lo tanto, no se puede garantizar el derecho a la salud de los migrantes sin su plena inclusión social, económica y cultural en la sociedad. Aunque las políticas de salud para migrantes abordan estrategias específicas para garantizar su acceso efectivo a los servicios de salud, el aseguramiento y los enfoques diferenciales, lo cierto es que la salud de esta población depende en gran medida de determinantes sociales, como la educación, el empleo formal y el ingreso. Estos factores van mucho más allá del sector salud y están condicionados por el contexto de las políticas migratorias ^5^. En consecuencia, las políticas migratorias pueden tener un impacto más significativo en la salud de los migrantes que las propias intervenciones en salud y que las políticas específicas de migración y salud.

Estos nacionalismos, además, amenazan nuestra efectividad en salud global, no solo para las metas a largo plazo, sino también para responder a emergencias sanitarias. Si surgiera pronto una nueva emergencia de salud global, como el COVID-19, las capacidades de cooperación necesarias se verían aún más debilitadas por las tendencias nacionalistas, que no reconocen que los virus no distinguen fronteras ni órdenes geopolíticos. Esto representa un retroceso en el entendimiento de la salud como una responsabilidad compartida, ya que la respuesta a estas amenazas sanitarias globales no será efectiva sin la coordinación entre países y la inclusión de los migrantes bajo un enfoque de equidad ^6^. La aproximación global e incluyente no solo parece ser la más ética, sino que epidemiológicamente es la correcta, ya que, en una pandemia, ningún país estará seguro sin que todos lo estén, incluidos los migrantes.

En Colombia, donde hoy residen cerca de tres millones de migrantes venezolanos, los avances en su inclusión social desde el 2017 enfrentan actualmente retrocesos, debido a la postura que ha adoptado el gobierno actual frente a Venezuela. No se trata, en este caso, de discursos nacionalistas y xenofóbicos -aunque también los hemos tenido en Colombia- sino de cálculos geopolíticos que han ralentizado los procesos de regularización de los migrantes venezolanos, incrementado la estigmatización social y debilitado la política migratoria. Estas barreras representan las principales amenazas para garantizar el derecho a la salud de esta población vulnerable. A pesar de la diversidad interna de la población migrante, en Colombia persisten inequidades significativas entre migrantes y no migrantes, y entre distintos grupos de migrantes ^7^. Se han identificado brechas sustanciales en salud materna, el acceso efectivo a servicios de salud y la atención en salud mental, sexual y reproductiva, entre otros aspectos ^8^. En cuanto al acceso efectivo a la salud para migrantes, la primera barrera suele ser la regularización, la cual, según el país, condiciona en distintos grados el acceso a determinados servicios de salud ^5^. Sin embargo, incluso entre migrantes con estatus regular, persisten obstáculos como el desconocimiento de las rutas de atención, la falta de enfoques diferenciales adaptados a sus necesidades, las barreras administrativas y, en algunos casos, la estigmatización por parte de los profesionales de la salud ^9^. Reducir estas desigualdades requiere la implementación y el fortalecimiento de programas con enfoques diferenciales, pero estos esfuerzos quedarán limitados sin una política migratoria integral y sostenida.

En una época de creciente nacionalismo, en la que las luchas por los derechos humanos están en constante amenaza, es fundamental rechazar la demagogia y no subordinar los derechos de los migrantes a los intereses geopolíticos particulares. Independientemente de la postura política, existe un imperativo ético: garantizar los derechos de los migrantes, incluido el de la salud. Sin embargo, este desafío no puede ser abordado exclusivamente por el sector salud, requiere una aproximación intersectorial y un compromiso colectivo para construir sociedades más inclusivas y justas.

El sistema de Naciones Unidas, nacido en la esperanza de la posguerra, tiene muchas fallas y problemas de efectividad, pero representa -con todos sus defectos inherentes a las instituciones humanas- un esfuerzo notable por fomentar el reconocimiento de los efectos positivos de la migración y por proteger los derechos de los migrantes. Actualmente, como consecuencia de las decisiones del nuevo gobierno de los Estados Unidos, el financiamiento de la Organización Internacional para las Migraciones enfrenta su peor momento, lo cual puede llevar a un incremento de la morbimortalidad de los cientos de miles de personas beneficiadas en el mundo con sus programas ^10^.

En el contexto donde los discursos nacionalistas ganan fuerza, el hablar de los derechos humanos de los migrantes puede parecer impopular e incluso ser objeto de burlas por parte de quienes niegan su condición de sujetos de derechos. Sin embargo, cuanto más fuerte sea el ruido de la exclusión, más urgente será hablar con claridad, determinación y sensatez.
